# Enzymatic Hydrolysis of Textile and Cardboard Waste as a Glucose Source for the Production of Limonene in *Escherichia coli*

**DOI:** 10.3390/life12091423

**Published:** 2022-09-13

**Authors:** Žiga Zebec, Mojca Poberžnik, Aleksandra Lobnik

**Affiliations:** 1 Faculty of Mechanical Engineering, University of Maribor, Smetanova Ul. 17, 2000 Maribor, Slovenia; 2 IOS d.o.o., Beloruska Ul. 7, 2000 Maribor, Slovenia

**Keywords:** cotton/cardboard (CC) waste, enzymatic hydrolysis, glucose juice, synthetic biology, limonene

## Abstract

Cellulose containing textiles (cotton) and cardboard/carton waste represent a large reservoir of untapped organic carbon. These wastes have enormous potential for use as carbon feedstock in industrial biotechnological processes. Essentially, cotton/cardboard (CC) waste is pure cellulose (with some additives) in the form of polymerised glucose consisting of β-(1→4)-linked D-glucose subunits. One of the largest and most diverse classes of natural chemicals that can be produced from glucose are terpenes with a wide range of applications as flavours, fragrances, pharmaceuticals, biopesticides, and biofuels. Here we have investigated the bioconversion of CC waste into the exemplary terpene limonene as a proof of concept. Six different CC waste streams were enzymatically hydrolysed and used to produce limonene using the *Escherichia coli* (*E. coli*) microbial cell factory. The D-glucose content in the CC hydrolysate (glucose juice) was determined and then metabolised by *E. coli* via a manipulated heterogeneous biolipid synthesis pathway (the mevalonate pathway) to produce limonene. This study represents an important proof of concept for the production of terpenes from hydrolysed CC waste streams.

## 1. Introduction

Environmental pollution and global climate change are the greatest challenges facing humanity due to the industrialised production of materials. Cotton and cardboard (CC) materials are one of the biggest contributors to municipal solid waste. Both are made of cellulose, which makes them important potential substrates for recycling or reuse technologies that can be applied in most regions of the world [1,2,3]. In order to mitigate the negative impacts of pollution and global climate change caused by these waste streams, it is now important to find new strategies to recover and manufacture new products from these abundant waste materials [4,5].

Cotton based textiles are usually produced in large quantities, using a large amount of chemicals to process raw materials (pre-consumer pollution), form fibres, develop, and produce different types of fabrics up to finished products [6]. In Spain, for example, textiles represent about 4% of total municipal solid waste [1], while globally it is about 5% [7]. To reuse or recycle textiles [8], they must be collected separately from household waste. After collection, the clothing must be separated into reusable and non-reusable clothing. Germany is one of the pioneers in the collection and sorting of textiles where 75% of textile waste is collected separately [9]. Even though the separate collection and sorting of used textiles requires labour and energy, every kilogramme of new cotton replaced by used material saves up to 65 kWh of energy [10]. European directives 2020 and the EU Textile Strategy 2021 promote the reuse and recycling of textile waste due to the high level of pollution during the manufacturing process [11].

Cotton-containing fabrics are made from cellulose, which consists of β (1→4)-linked D-glucose. Globally, more than 20 million tonnes of cotton fibre are produced per year and only about 15% is reused or recycled, while 85% of textile waste ends up in landfills as it is essentially unusable for fibre production [6,12]. In the EU, about 20% of the 5.6 megatons (Mt) of textile waste was reused or recycled in 2013 [13]. This cellulosic waste is an important source of stored organic carbon with untapped potential as a substrate for the production of “added value” natural products [14].

Cardboard waste is another important source of cellulose, mostly used as a packaging material, accounting for 1–6% of municipal solid waste, depending on the country and micro location [1,2]. However, in developed countries, cardboard is usually collected separately from general municipal solid waste by general waste collectors [1]. The simplest way to recycle cardboard is composting [15] together with other organic material. More sophisticated recycling methods have been developed recently, allowing the production of value-added natural chemicals and renewable energy in the form of hydrogen [16]. However, the proportion of cardboard that can be processed in this co-digestion process is relatively small compared to food waste, and the choice of value-added chemicals is limited to a few natural compounds [16].

In order to recycle waste containing CC material in a flexible manner, cellulases are employed for the enzymatic hydrolysis of cellulosic textile waste [17]. Commercially available cellulases are available from Novozymes as an enzyme blend consisting of Cel7A, Cel7 B, and beta-glucosidase under the name Cellic CTec2 (Novozymes) [18]. These enzymes can act on cellulose by binding to different sites on the β(1→4)-linked D-glucose that forms cellulose. Each of the enzymes included in the Cellic Ctec2 enzyme blend has its own function. Cel7A is an exoglucanase that acts on the cellulose polymer to create notches that expose reducing and non-reducing ends of cellulose. Cel7 B is an endoglucanase that can act on a nicked cellulose structure to produce cello-oligosaccharides and cellobiose (two D-glucose subunits). The hydrolysis of cellulose is completed by β-glucosidase, which can cleave cellobiose into two D-glucose molecules, completing the process that leads to the formation of glucose juice [18,19].

Glucose can be used as the main carbon source for the microbial production of biofuels and other value-added biochemicals, biofuels, and monomers for the production of advanced biological materials [20]. The most common process is the conversion of glucose into bioethanol through fermentation [19,21]. However, dissolved CC waste could be converted into value-added natural chemicals using synthetic biology approaches. One class of natural chemicals that can be produced with glucose are monoterpenes [22]. These can be produced via two different metabolic pathways: the methylerythritol phosphate isoprenoid pathway (MEP) and the mevalonate pathway (MVA) [23]. Several classes of monoterpenes have been produced via the MVA pathway [24]. The most common monoterpene produced using synthetic biology is (S)-limonene and its derivative perillyl alcohol, which were produced in *E.coli* [25]. The nine genes required for the synthesis of limonene are encoded in a synthetic gene cluster (SGC), which also has all the required intrinsic genetic controls (codon-optimised genes, including ribosome binding sequence (RBS), promoters, terminator and etc.). All these features are present on the plasmid pJBEI-6410, which allows for the production of limonene in a culture volume of 5 mL [25,26,27]. With the same plasmid, up to 3.6 g/L limonene could be produced in an industrial setting [28].

Here we enzymatically hydrolysed various cellulosic waste streams and used them to produce glucose juice. The glucose juice was sterile filtered and used directly at appropriate concentrations as a substrate for the production of terpenes such as limonene. We produced limonene from all six glucose juices, which are the products of enzymatic hydrolysis of CC waste. Overall, this work demonstrates the potential applicability of this recycling concept with various complex substrates, even with composite wastes, to produce natural compounds such as limonene using microbial cell factories.

## 2. Materials and Methods

### 2.1. Composition of Waste Materials

**Figure 1:**
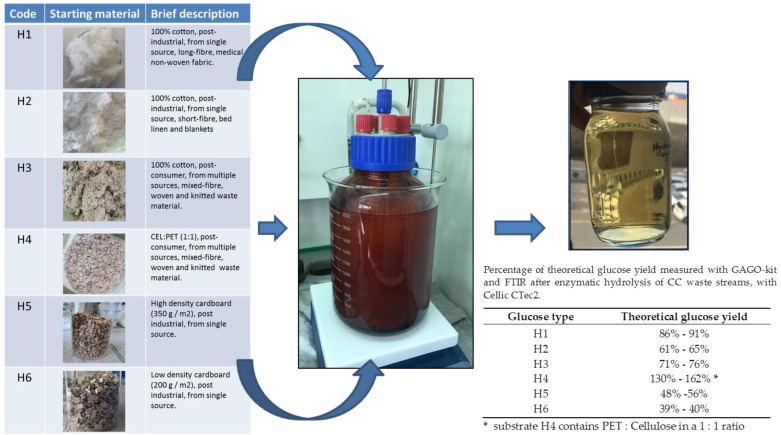
Schematic representation of the enzymatic hydrolysis of various CC containing wastes (H1 to H6) in an amber 2.5 L reactor, glucose juice (sterile filtered), and the percentage of theoretical yield.

Six different cellulose substrates/waste streams were selected for enzymatic hydrolysis (Figure 1). Substrate H1 is from post-industrial waste from a single source (Tosama, a company that develops, manufactures, and distributes hygiene and medical supplies) in the form of pure white, long-fibre, 100% cotton medical nonwoven fabric. Substrate H2 is also a post-industrial waste, but unlike H1, it consists of shorter cotton fibres with a less homogeneous structure and a wider distribution of fibre lengths. H2 is also 100% cotton, non-coloured post-industrial waste, but comes from a different source (Odeja, the leading Slovenian manufacturer of high-quality quilted textile products, e.g., bed linen and blankets). The other substrates H3 and H4 came from post-consumer textile waste. The old clothes were sorted according to their raw material composition; all metal and plastic parts were removed before mechanical shredding (piece size 8 mm). The material used in H3 consisted of 100% cotton, woven, and knitted textile material, while H4 consisted of polyester/cotton (PET/CELL) textile waste blends with an average PET:CELL ratio of 1:1. Substrates H5 and H6 were pure cardboard from the paper processing industry, unbleached and unprinted industrial waste cut into 8–10 mm pieces. They differ slightly in structure and density, i.e., 350 g/m^2^ (H5) and 200 g/m^2^ (H6).

### 2.2. Enzymatic Hydrolysis

Cellulosic waste material was weighed to 120 g, pre-treated mechanically and chemically by autoclaving (at 131 °C and 1.79 bar) in 1% NaOH solution (*w*/*v*) to decolourise and open/break up the fibres before enzymatic hydrolysis. After pre-treatment, the material was rinsed with deionised water and adjusted to pH 6 with glacial acetic acid. The 2.5 L amber glass reactor was filled with 1.8 L 0.1M sodium acetate buffer (pH 8) and heated to 50 °C. The pre-treated material was added to the 2.5 L amber glass reactor (Figure 1) with 50 g of substrate material per 1 L of buffer. The first sample (T_0_) was taken before the addition of the enzymes, while sample T_1_ was taken immediately after the addition of 10 mL of the available enzyme blend Cellic CTec2 from Novozymes, which contains a mixture of glycoside hydrolases (cellobiohydrolase I (Cel7A), endoglucanase I (Cel7B), and β-glucosidase) capable of degrading cellulose [18]. Enzymatic degradation was carried out at 50 °C, 250 rpm (adapted to the material) for a maximum of 8 days (Figure S1).

### 2.3. Glucose Quantification

Samples were taken hourly for the first 24 h and then twice daily to quantify the glucose produced during enzymatic hydrolysis using the GAGO kit (Sigma) according to the manufacturer’s instructions. Briefly, a GAGO kit uses the glucose-specific enzyme glucose oxidase to oxidise glucose to gluconic acid and hydrogen peroxide. Hydrogen peroxide reacts with o-dianisidine in the presence of peroxidase to form a stable, coloured product that can be measured spectrophotometrically at 540 nm. To accurately determine the glucose concentration during the enzymatic hydrolysis of waste, a five-point standard curve (from 0 µg to 80 µg) was established with pure glucose. To be within the test range, a dilution factor for the enzymatically hydrolysed glucose juice must be determined before the samples can be accurately processed.

In addition, the final glucose concentration after enzymatic hydrolysis (Table 1) was determined using Fourier transform infrared spectroscopy (FTIR). In FTIR, attenuated total reflectance (ATR) using a single reflectance diamond crystal was used to record the infrared spectra (IR) of the samples under investigation. The spectra were recorded using a PerkinElmer spectrometer FT-IR (Spectrum Two). Each sample spectrum was recorded with eight scans per spectrum in the wavenumber range from 4000 to 400 cm^−1^. For calibration, the spectrum of a blank was recorded with 0.1 M sodium acetate at room temperature (about 23 °C), followed by the standards. The nominal recording resolution of 4 cm^−1^ was used to study the absorption peak of glucose (at 1034.68 cm^−1^) with the base at 1185.31 cm^−1^. The ATR element was cleaned with a soft paper towel and dried before the spectra were recorded again. The individual spectra of blank, standards, and samples were transferred to Spectrum Quant software for analysis and quantification. Lambert-Beer’s law was applied and a five-point standard curve with pure glucose (from 0 g/L to 50 g/L) was used.

After 8 days of enzymatic hydrolysis, the reaction was stopped, regardless of glucose concentration, and the remaining hydrolysed material was sterile filtered with a 0.25 µm filter and stored at 4 °C for later use (Figure 1).

### 2.4. Growth Curves with Media Containing Glucose Juice

*E. coli* BL21 transformed with pJBEI-6410 were isolated from the LB plate and then grown in 5 mL selective medium (LB or M9), supplemented with glucose juice. All cultures were grown and treated as described below in the limonene production experiment. Cell density was determined spectrophotometrically by measuring absorbance at 600 nm (optical density, OD). The number of cells present in the media was determined over a period of 3 days. Samples were taken every 30 min for the first 5 h and then after 8.5 h, 25.5 h, 30 h, 47 h, and 72 h. Three separate measurements were taken per time point.

### 2.5. Limonene Production and Isolation

pJBEI-6410 [25] was used for the production of limonene. Cells were transformed with the pJBEI-6410 in chemically competent *E. coli* DH-10β in LB media with ampicillin. The plasmid was propagated, extracted, and transformed back into *E. coli* BL21 and plated onto selective LB plates. Three individual colonies were isolated from the LB plate and later grown in 300 µL of non-selective LB medium in a 1.5 mL plastic tube for 3 h. After 3 h, 40 µL of the cell culture was used to inoculate 5 mL of M9 or LB media supplemented with the appropriate glucose juice as a carbon source. The final glucose concentration was set to 0.4%, calculated from the concentrations estimated by the GAGO kit (Table 1). To increase the cell density of the 5 mL cell culture, it was grown at 30 °C and 200 rpm for 3 h. The production of limonene was induced by the addition of 25 µM isopropyl-β-D-thiogalactoside (IPTG), while limonene was entrapped in 10% (*v*/*v*) organic overlay (dodecane).

Cells were grown at 30 °C and 200 rpm for 72 h before the organic overlay was extracted and the optical density (OD) of the cell culture was measured. The extracted overlay was separated by centrifugation for 10 min at 15,000 rpm at 4 °C and dried with MgSO_4_ to remove any water. Another centrifugation step was added for 5 min at 4 °C and 15,000 rpm to sediment the MgSO_4_. Finally, the dried overlay was decanted and an equal volume of ethyl acetate containing 0.1% sec-butylbenzene was added. The later chemical sec-butylbenzene serves as an internal standard to control the injection volume in gas chromatography (GC).

### 2.6. Product Quantification with Gas-Chromatography (GC)

Volatile extracts of *E. coli* trapped in the organic overlay (dodecane) were analysed by gas chromatography (GC) on an Agilent Technologies 7890A GC system equipped with a FID detector and a 7693 autosampler. A DB-WAX column (30 m; 0.32 mm; 0.25 μm film thickness; JW Scientific) was used to separate the compounds. The injector temperature was set at 220 °C with a split ratio of 10:1 (1 μL injection). The carrier gas was hydrogen with a flow rate of 1.5 mL/min and a pressure of 10 psi. The following oven programme was used: 50 °C (0 min hold time), ramp to 200 °C at 5 °C/min (0 min hold time), and ramp to 240 °C at 20 °C/min (1 min hold time). The FID detector was held at a temperature of 280 °C using a hydrogen flow of 30 mL/min. Limonene was identified using authentic standards (Sigma, CAS: 5989-54-8). The product was quantified with authentic standards, using experimentally determined peak area in relation to the peak area of the co-injected internal standard (0.1% sec-butylbenzene).

## 3. Results

All six waste streams from CC were hydrolysed (Figure 1) under the same conditions, with substrates H1, H3, and H4 yielding an average of about 40 g glucose per L of reaction, while substrates H2, H5, and H6 had a lower glucose concentration of 20–30 g/L (Table 1).

All six waste streams from CC were hydrolysed under the same conditions, with substrates H1, H3, and H4 yielding on average about 40 g of glucose per L of reaction with a higher percentage of theoretical yield according to GAGO, while substrates H2, H5, and H6 had a lower glucose concentration of 20–30 g/L and a low percentage of theoretical yield (Table 1). The onset of the reaction of all six substrates started with an exponential increase of glucose in the first hours of treatment, but only H1 followed a similar hydrolysis pattern, as in the project RESYNTEX (Figure S1). Substrate H4 showed a rather unusual oscillation of glucose concentration, probably due to its composition (the presence of PET), but resulted in a similar final concentration. The enzymatic degradation of substrates H2 and H3 was rather slow, but the final percentage of theoretical yield was 61% and 76%, respectively. Substrates H5 and H6 behaved very similarly in enzymatic hydrolysis, but only achieved about 20–25 g/L glucose and a low percentage of the theoretical yield under the conditions tested.

Growth media containing glucose juice from various CC waste streams were investigated for their suitability as an alternative carbon source to pure glucose. Cell density was determined spectrophotometrically by measuring optical density (OD), and the number of cells present in the media was determined over a period of 3-days (Figure S2). In all experimental arrangements, an OD of 2 was reached after 3 days of growth, except for H3 in M9 media, where an OD of about 1 was reached. The highest OD was achieved with substrate H4 in LB media (OD = 2.5). Growth curves for substrates H5 and H6 were not generated because turbidity developed after the addition of these two substrates, preventing the adequate measurement of OD.

**Figure 2:**
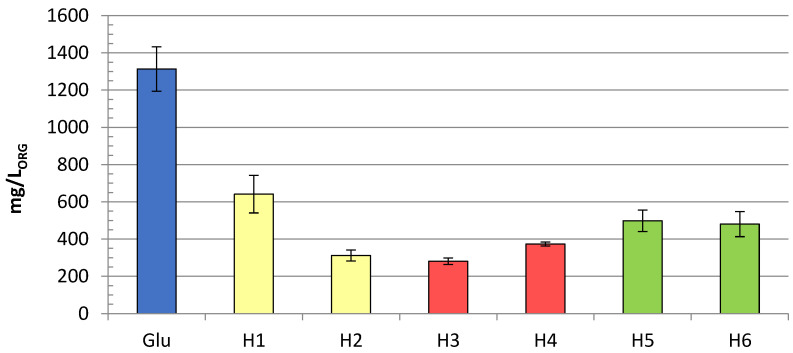
Production of limonene in mg per L of organic phase in *E. coli* BL21 transformed with pJBEI-6410. M9 media was supplemented with CC waste glucose juices as a glucose source. Error bars represent standard deviation with at least three biological replicates.

To test the production of limonene with glucose juice from different CC waste streams, pJBEI-6410 was transformed into *E. coli* BL21. Individual colonies were isolated and tested, as described above. Limonene production was observed with all substrates (Figure 2). With substrate H1, the yield was about 650 mg limonene per litre of the organic phase. With the glucose juice of H2 and H3, only about 300 mg/L could be produced, while with H4, 370 mg/L were produced. With the cardboard containing substrates H5 and H6, the production was over 450 mg per L.

The highest production titre in our experiment with LB media was achieved with pure glucose, with about 1100 mg/L compared to only about 200 mg/L with H1, while the production of H2, H4, H5, and H6 was detected at very low levels (below 20 mg/L), and no production was detected with substrate H3 (Figure S3).

## 4. Discussion

To investigate the range of materials that can be enzymatically hydrolysed, we have selected six different types of CC waste materials belonging to three different categories (H1–2, H3–4, and H5–6). In the first category, both substrates (H1 and H2) were post-industrial wastes consisting of pure cotton without coloration. Substrate H1 was chosen as a starting point to show that the production of terpenes with glucose juice is possible. As expected, this material was efficiently degraded by enzymatic hydrolysis and reached a final glucose concentration of 43 g/L and a final percentage of the theoretical yield of 86%, according to GAGO. In contrast, enzymatic hydrolysis of H2 only resulted in a final glucose concentration of about 30 g/L, which is only about 65% of the theoretical yield (Table 1). Overall, the hydrolysis was much slower with substrate H2, even though both H1 and H2 are non-coloured 100% cotton. Surprisingly, the shorter fibre length and less homogeneous structure of H2 seem to have influenced the efficiency of enzymatic hydrolysis. Similar effects, that shorter fibres are less degraded in enzymatic hydrolysis, have been observed elsewhere in the enzymatic hydrolysis of short pulp fibres [29].

The second category of substrates (H3 and H4) is post-consumer waste from discarded textiles (clothing). Substrate H3, which is genuine woven and knitted textile waste, contains 100% cellulose. The enzymatic hydrolysis of H3 was slower compared to the other substrates, but still reached a final concentration of 40 g/L and a theoretical yield of 75% was reached after 8 days of treatment (Table 1). Substrate H4 contains polyethylene terephthalate (PET) and cotton in a 1:1 ratio, which does not seem to have a negative effect on enzymatic hydrolysis. Enzymatic hydrolysis of H4 resulted in a final concentration of about 40 g/L after 8 days of treatment (Table 1) with a percentage of theoretical glucose yield of over 130–162%, as only 25g of glucose per L of buffer was expected. The oscillation of the glucose concentration was also not expected (Figure S1). One reason for this behaviour could be the polyethylene glycol (PEG) contained in this material, as PET is used as a conventional approach to detach the enzymes from the cellulose residues [18,30] and which could prevent accurate measurement with glucose oxidase (GAGO). Another reason for these variations in glucose concentration could be the mixing of the waste material in the reactor, which could have affected the degradation efficiency and glucose measurement [29]. However, the glucose juice used for production was also measured with FTIR, which showed a lower glucose concentration for H4, suggesting that the estimation of glucose concentration for mixed composition substrates may not be as straightforward as expected [19]. This behaviour of H4 was not further investigated. The final category is cardboard waste H5 and H6, which differ slightly in terms of construction and mass per unit area. Both materials consisting of cardboard were not well degraded by enzymatic hydrolysis, with 48% and 42%, respectively. The reaction was comparable to all other substrates within the first 24 h after treatment, but did not reach more than 25 g/L after 8 days of treatment. The low yield of glucose could be related to the additives used during the pulping process in the production of cardboard [31].

Overall, all six materials of the CC waste streams could be digested with the Cellic CTec2 enzyme mixture and reached a similar final concentration (40 g/L), as observed in the RESYNTEX project and elsewhere [19,29], except for material H4, H5, and H6. However, only post-industrial waste (H1) could be digested in a similar way as previously observed for pure cellulose material [29], suggesting that process optimisation is required for each type of CC waste stream material. Both methods for quantifying glucose, GAGO and FTIR, which have already been used to measure glucose concentration [32], have shown similar results in terms of glucose concentration in glucose juices, except for H4 (Table 1). A solution to the problem of substrate diversity could be to mix different CC waste streams into mixtures that have a specific chemical composition and fibre structure. In this way, the optimisation of the enzymatic degradation processes would only have to be carried out once for each substrate mixture. In order to improve this process as a whole and make it more ecologically compatible [19], a new enzyme mixture was developed—Cellic CTec3, which can also be coupled with enzyme recycling [33].

To mimic cell culture growth during the production process, *E. coli* BL21 transformed with pJBEI-6410 was grown in M9 or LB media, supplemented with pure glucose or one of the glucose juices as a substitute for pure glucose. All cultures grew under the conditions tested, most reaching an OD of 2. Interestingly, cell growth during the production process using pure glucose as a carbon source appears to occur in two separate exponential growth phases (Figure 2). The maximum OD was 2.5 with H4 in LB media, but this did not result in a higher limonene yield (Figure S3). These results show that higher cell density does not necessarily mean high limonene production, which has also been observed by others [25]. Growth curves could not be generated for substrates H5 and H6 because the addition of these two substrates resulted in turbidity of the medium, which would result in an inaccurate measurement of OD.

The resulting glucose juices with the determined glucose concentrations were added to the production media of *E. coli*, to a final concentration of 0.4%, which was calculated using the results obtained with the GAGO kit (Table 1). With glucose juice H1 as a carbon source, about 650 mg/L of limonene was produced, which is about 50% of the production with pure glucose (1300 mg/L) in M9 media (Figure 2). With substrates H2 and H3, only 300 mg/L could be produced in M9 media. While the lower yield with the post-consumer cellulose waste H3 was somehow expected, this was not the case with H2, as H2 is also made of 100% cotton, which is non coloured, and, like H1, comes from industrial sources. Since H1 is used as a medical material, it is likely that less additives need to be used in the production of H1 and a strict manufacturing process [34] needs to be followed to minimise impurities during the manufacturing process. This suggests that M9 media enriched with pre-consumer glucose juice H1 contain fewer impurities than glucose juice from waste H2, H3, and H4, which negatively affect limonene production but do not inhibit growth (Figure S2). The yield with H4 was about half (370 mg/L) of the yield with H1 and 25% of the limonene produced with pure glucose. The production of limonene per unit of glucose with substrate H4 is not reliable because the quantification of glucose by GAGO and FTIR was overestimated (Table 1). Nevertheless, this shows that the presence of PET has no negative influence on limonene production. Limonene production with cardboard materials H5 and H6 was higher than the yield with H2, H3, and H4, although the M9 production medium became turbid after the addition of glucose juice. However, production was not affected by the resulting turbidity and about 500 mg/L limonene could be produced, more than with the post-consumer textile wastes (H2, H3, and H4).

In general, the limonene yield was lower with LB media, regardless of the glucose source. The maximum yield was about 1100 mg/L with pure glucose, while it was limited to about 200 mg/L with H1 (Figure S3). With all other substrates, the yield was less than 20 mg/L, and no limonene was produced with glucose juice H3 in LB media. Similar behaviour regarding production of limonene in minimal M9-media compared to rich media (LB) was observed also by others [28]. An important factor in using glucose juice as a substrate for limonene production is that sterile filtered glucose juice was stable at 4 °C for more than 12 months. Similar amounts of limonene can be produced from fresh glucose juice and stored glucose juice (Figure 2). In addition, other terpenes, such as linalool, can also be produced from glucose juice (data not shown).

Limonene synthase (LS), encoded on plasmid pJBEI-6410, also produces other monoterpenes, by-products, such as citronellol, nerol, geraniol, and farnesol [24,35], which were not further investigated in this study. The metabolic remodelling of limonene produced by LS has been published elsewhere [35]. Since limonene also has antibacterial activity against *E. coli* at certain concentrations, this is one of the limitations of producing higher titres of limonene [36,37,38] and needs to be addressed to make this technology useful for recycling. For instance applications such as limonene nanoemulsions can even enhance the antimicrobial effect by limiting biofilm formation, curli and EPS production [39]. One of the toxic intermediates in the synthetic production of limonene by *E. coli* is limonene hydrogen peroxide, the formation of which can be limited by a mutation in *ahpC*, which encodes alkyl hydrogen peroxidase [36].

Compared to other attempts to produce limonene by synthetic biology using the plasmid pJBEI-6410, we obtained similar amounts of limonene [25]. Production can be further increased through optimisation measures, such as principal component analysis of proteomics (PCAP), which increased the production of limonene by 40% [26]. Further improvements were achieved by integrating the SGC encoded on the plasmid pJBEI-6410 into the chromosome of *E. coli* to improve the genetic stability of the production strain and increase the titres that can be produced [27].

## 5. Conclusions

We have shown here that the direct conversion of waste into glucose juice and further into limonene or other terpenes is feasible on a laboratory scale in *E. coli*. In order to be able to apply our biotechnological recycling strategy with the production of natural chemicals using synthetic biology on an industrial scale, this technology must be economically viable. To achieve this goal, different strategies can be applied: (i) the production of higher titres of value-added chemicals [28], (ii) the production of natural chemicals that have higher value in the market [40], or (iii) the combination of enzymatic hydrolysis of cellulose and the production of value-added chemicals in one organism or designing a production consortium of microbes [41], where one microbe can degrade the substrate and produce building block chemicals for its partner for the production of value-added chemicals. This approach, in combination with mechanical, chemical, and enzymatic treatment methods [17], can possibly provide an economical and environmentally friendly way to treat waste and produce value-added chemicals through synthetic biology.

## Figures and Tables

**Table 1:** Final glucose concentrations of glucose juice measured with GAGO-kit and FTIR after enzymatic hydrolysis of CC waste streams, using Cellic CTec2. The percentage of the theoretical yield is given in brackets.

Glucose Type	Glucose Conc. g/L GAGO ^1^	Glucose Conc. g/L FTIR ^2^
H1	43.23 ± 0.91 (86%)	45.64 ± 1.18 (91%)
H2	30.91 ± 0.56 (61%)	32.58 ± 0.93 (65%)
H3	38.33 ± 0.36 (76%)	35.90 ± 0.89 (76%)
H4	40.65 ± 0.12 (162%) *	32.52 ± 0.93 (130%) *
H5	24.14 ± 0.44 (48%)	27.90 ± 0.78 (56%)
H6	21.04 ± 0.81 (42%)	19.92 ± 0.83 (39%)

^1^ standard deviation of three technical replicates; ^2^ prediction error of eight scans; * PET:CELL = 1:1.

## Data Availability

All data obtained in this study are included in this published article and its supplementary information. However, the raw data analysed are available on request from the corresponding author.

## Supplementary Materials

The following supporting information can be downloaded at: https://www.mdpi.com/article/10.3390/life12091423/s1. Figure S1. Measurements of glucose concentrations with the GAGO-kit during the enzymatic hydrolysis of CC waste streams, with Cellic CTec2. Figure S2. Growth curves of the production strains in different media (LB or M9), supplemented with the corresponding glucose juice, from enzymatic hydrolysis. The horizontal axis represents the time in minutes, while the vertical axis shows the optical density (OD) measured at 600nm. Dashed lines represent the growth curves of the limonene production strain grown on glucose. The markers represent the average of three technical replicates. Figure S3. Production of limonene in mg per litre of organic phase in *E. coli*-BL21 transformed with pJBEI-6410. M9 media or LB media is supplemented with glucose juices from CC waste. Error bars represent standard deviation with at least three biological replicates. Table S1. Average (Ave.) production of limonene in mg per litre of organic phase in *E. coli*-BL21 transformed with pJBEI-6410. M9 media or LB media is supplemented with CC waste glucose juices. Standard deviation (SD) is calculated with at least three biological replicates.
