# Supplementary material for: Enzymatic Hydrolysis of Textile and Cardboard Waste as a Glucose Source for the Production of Limonene in Escherichia coli
Source: Life (Basel). 2022 Sep 13;12(9):1423. doi: 10.3390/life12091423 (PMC9500893; doi:10.3390/life12091423)
Supplement: Supplementary file 1 [file life-12-01423-s001.zip › life-1841771-supplementary.pdf]

Supplementary Material

# Enzymatic hydrolysis of textile and cardboard waste as a glucose source for the production of limonene in *Escherichia coli*

**Žiga Zebec [1,*], Mojca Pobrežnik [2] and Aleksandra Lobnik [1]**

* corresponding author

[1] University of Maribor, faculty of mechanical engineering, Smetanova ul. 17, Maribor, Slovenia

[2] IOS d.o.o., Beloruska Ul. 7, Maribor, Slovenia

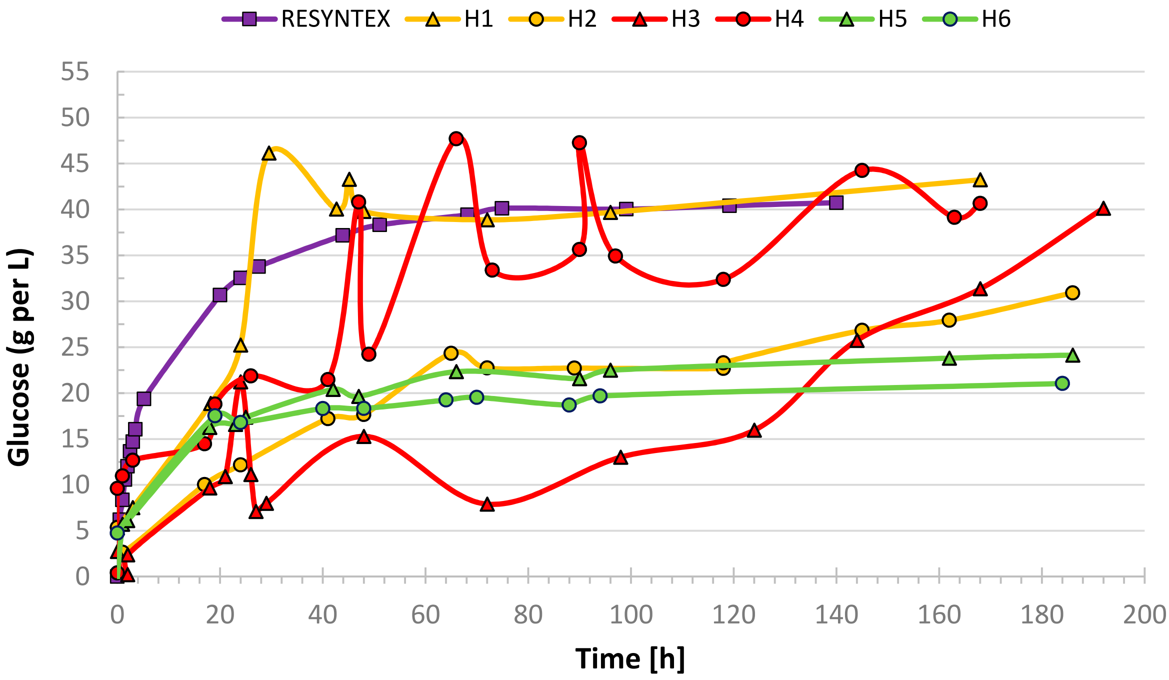


**Figure S1.** Measurments of glucose concentrations with the GAGO-kit during the enzymatic hydrolysis of CC waste streams, with Cellic CTec2.

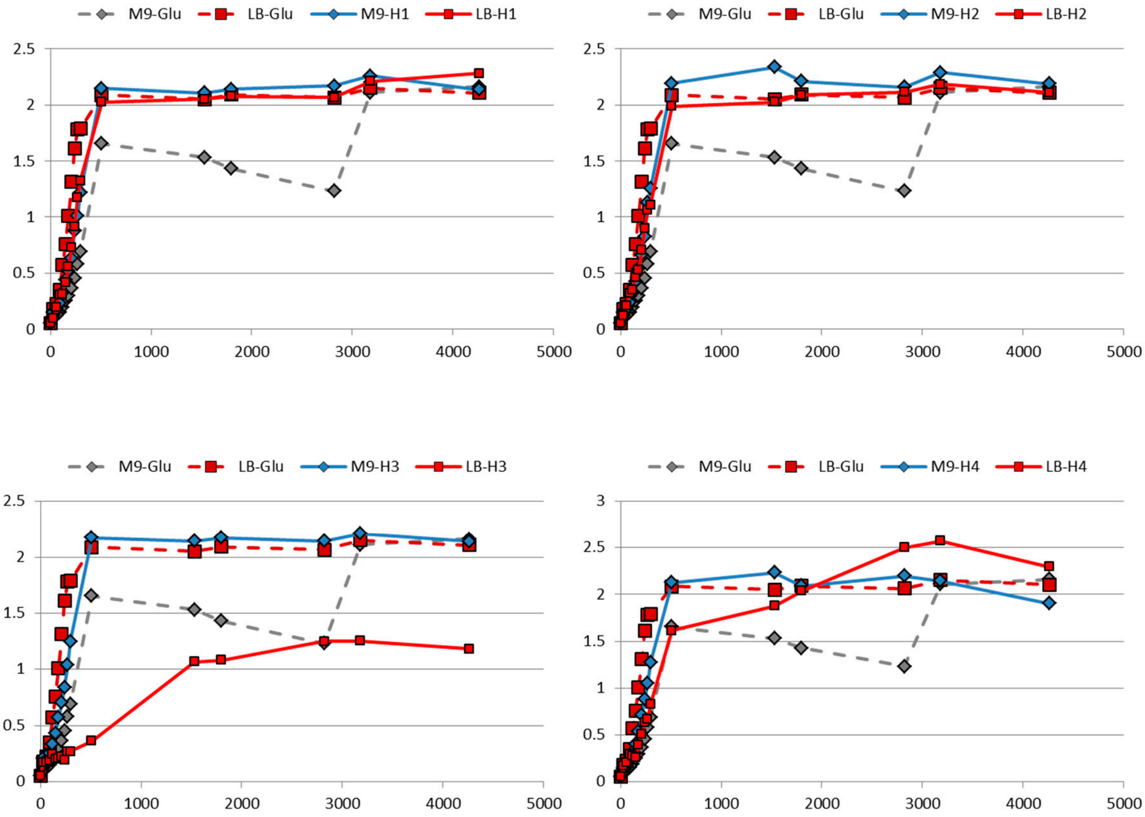


**Figure S2.** Growth curves of the production strains in different media (LB or M9), supplemented with the corresponding glucose juice, from enzymatic hydrolysis. The horizontal axis represents the time in minutes, while the vertical axis shows the optical density (OD) measured at 600nm. Dashed lines represent the growth curves of the limonene production strain grown on glucose. The markers represent the average of three technical replicates.

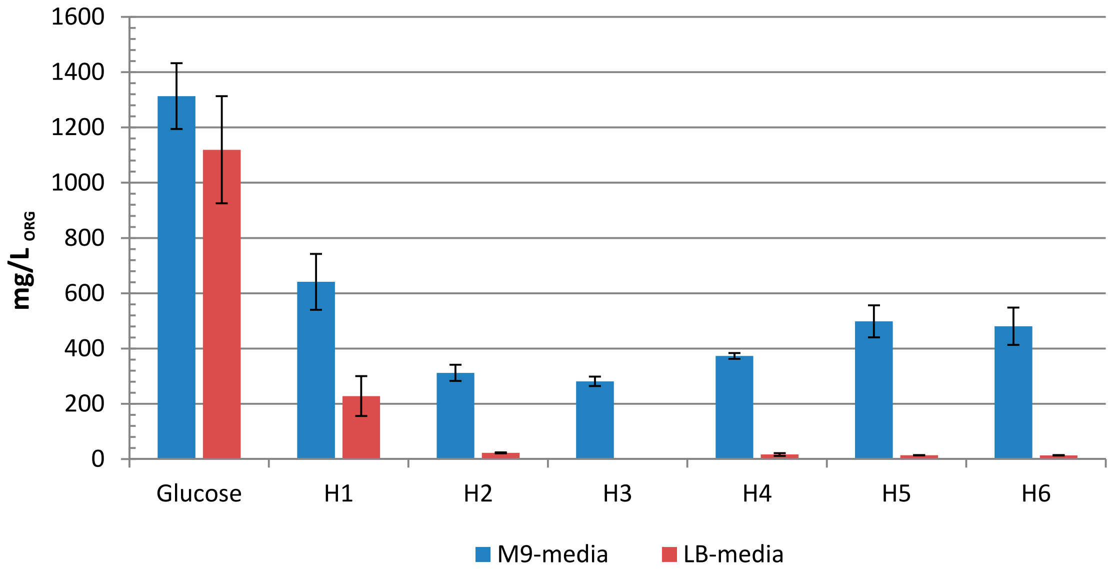


**Figure S3.** Production of limonene in mg per litre of organic phase in *E. coli*-BL21 transformed with pJBEI-6410. M9 media or LB media is supplemented with glucose juices from CC waste. Error bars represent standard deviation with at least three biological replicates.

| Media type | Glucose type | Ave. mg/L | SD mg/L | Ave. mg/L*OD | SD mg/L*OD |
|---|---|---|---|---|---|
| LB | Glucose | 1119.33 | 193.59 | 668.76 | 167.12 |
| LB | H1 | 228.00 | 72.08 | 239.24 | 101.40 |
| LB | H2 | 22.67 | 1.70 | 16.93 | 3.09 |
| LB | H3 | [redacted] | | | |
| LB | H4 | 16.60 | 5.05 | 17.82 | 5.99 |
| LB | H5 | 13.93 | 0.70 | 13.80 | 0.70 |
| LB | H6 | 13.60 | 0.72 | 13.47 | 0.71 |
| **Media type** | **Glucose type** | **Ave. mg/L** | **SD mg/L** | **Ave. mg/L*OD** | **SD mg/L*OD** |
| M9 | Glucose | 1313.33 | 119.06 | 596.78 | 79.92 |
| M9 | H1 | 641.27 | 100.91 | 435.51 | 220.80 |
| M9 | H2 | 312.07 | 29.26 | 135.25 | 11.91 |
| M9 | H3 | 281.23 | 17.21 | 121.83 | 7.54 |
| M9 | H4 | 373.4 | 10.34 | 294.46 | 53.57 |
| M9 | H5 | 498.40 | 57.71 | 156.45 | 16.47 |
| M9 | H6 | 480.60 | 67.56 | 143.95 | 18.48 |

**Table S1.** Average (Ave.) production of limonene in mg per litre of organic phase in *E. coli*-BL21 transformed with pJBEI-6410. M9 media or LB media is supplemented with CC waste glucose juices. Standard deviation (SD) is calculated with at least three biological replicates.
